# The relationships between lower-extremity dexterity and fall risk, fear of falling, and walking skills in People with Parkinson’s disease

**DOI:** 10.1007/s11845-026-04411-1

**Published:** 2026-04-27

**Authors:** Çağrı Gülşen, Ahmet Onur Keskin, Elvan Özcan Gülşen, Fatih Söke

**Affiliations:** 1 Faculty of Health Sciences, Department of Physiotherapy and Rehabilitation, Eskişehir Osmangazi University, Eskişehir, Turkey; 2 Department of Neurology, Eskişehir City Hospital, University of Health Sciences, Eskişehir, Turkey; 3 Yunus Emre Vocational School, Department of Health Care Services, Program in Geriatric Care, Anadolu University, Eskişehir, Turkey; 4 Gulhane Faculty of Physiotherapy and Rehabilitation, University of Health Sciences, Ankara, Turkey

**Keywords:** Fall risk, Fear of falling, Lower-extremity dexterity, Parkinson’s disease, Walking

## Abstract

**Background:**

The link between lower extremity dexterity and fall risk among people with Parkinson’s disease (PwPD) needs to be explore.

**Objective:**

To examine the relationships between lower-extremity dexterity and fall risk, fear of falling (FoF), and walking skills in PwPD.

**Methods:**

Thirty PwPD were included in the study. Fall risk was determined by the number of falls in the last six months. The Lower-Extremity Dexterity Task (LEDT) for lower-extremity dexterity; the Activities-Specific Balance Confidence Scale (ABC) for FoF; 10-Meter Walk Test (10MWT), the Timed Up and Go Test (TUG), the Figure-of-Eight Walk Test (FEWT), and the Three-Meter Backward Walk Test (TMBWT) for walking skills were used.

**Results:**

Fallers were significantly worse than non-fallers in terms of disease duration, H&Y, UPDRS-3, LEDT Most affected side (MAS), LEDT Least affected side (LAS), ABC, 10MWT, TUG, FEWT and TMBWT (*p* < 0.05). The LEDT-MAS and LEDT-LAS side were highly correlated with ABC, TUG, and FEWT and moderately correlated with H&Y, UPDRS-3, 10MWT, TMBWT (*p* < 0.05). The LEDT was a predictor of fall risk with an odds ratio of 1.204 (1.042–1.391) for MAS and odds ratio 1.245 (1.055–1.469) for LAS. The cut-off time of LEDT for best discriminating fallers from non-fallers was 26.23 s for MAS and 23.11 s for LAS.

**Conclusion:**

Lower-extremity dexterity is an independent predictor of fall risk in PwPD. Moreover, lower-extremity dexterity is related to FoF and walking skills in PwPD.

## Introduction

Parkinson’s disease (PD) is one of the fastest-growing neurological disorders worldwide, with symptoms such as bradykinesia, tremor, rigidity and postural instability [1]. These symptoms often affect the walking and balance functions of people with PD (PwPD) [2]. Therefore, PwPD lose their independence in activities of daily living and their quality of life decreases [3].

Lower extremity dexterity is the ability to dynamically control foot movements by coordinating the lower extremity joints. Lower extremity dexterity is critical for performing mobility tasks such as walking, running, and turning [4]. Additionally, lower extremity dexterity plays an important role in many activities of daily living, such as climbing stairs, stepping over obstacles, and driving [5]. However, it is reported that PD negatively affects lower extremity dexterity [6]. Consequently, decreased lower extremity dexterity can lead to problems.

Disorders in lower extremity functions cause walking problems in PwPD, independent of other symptoms [7]. Additionally, lower extremity dexterity and walking speed or functional mobility are related [4]. Moreover, lower extremity dexterity is associated with the risk of falling in multiple sclerosis [8]. There is a lack of studies exploring the link between lower extremity dexterity and fall risk among PwPD. Additionally, current evidence is insufficient to determine whether a relationship exists between lower extremity dexterity and fear of falling. Although previous studies in PwPD have identified associations between lower extremity dexterity and measures of walking speed and functional mobility, it is still unknown whether these associations extend to advanced gait tasks, such as curved and backward walking. Accordingly, this study aimed to examine the relationships between lower extremity dexterity, fall risk, fear of falling, and walking skills in PwPD.

## Methods

### Study design

This cross-sectional study was carried out at the Department of Neurology, Eskişehir City Hospital, between September and November 2025. The study was approved by the Ethics Committee of the Eskişehir City Hospital with decision number ESH/BAEK 2025/204. All participants provided written informed consent in accordance with the Declaration of Helsinki.

### Participants

A sample size calculation based on previous data [8] indicated that a minimum of 30 participants were required to achieve 80% statistical power with a 5% Type I error rate. Consequently, 30 PwPD were conveniently recruited from the Neurology Department of Eskişehir City Hospital.

All participants were: 1) diagnosed with PD according to current diagnostic criteria by an experienced neurologist [9]; 2) 40 years of age or older; 3) able to ambulate independently, with or without assistive devices; 4) in Hoehn and Yahr stage 1–3. PwPD were excluded from the study if they had: 1) any neurological disorder other than PD; 2) any orthopedic, musculoskeletal, vestibular, or other disorders that may affect the ability to walk; 3) undergone deep brain stimulation surgery.

### Outcome measures and procedures

All assessments were performed in “on” condition. The assessments were completed in a single session by an experienced physiotherapist in neurorehabilitation. The assessments were conducted in a random order. To minimize the effects of fatigue, three minutes of resting periods were given between each assessment. Most affected side was determined with patient self-report and the Unified Parkinson’s Disease Rating Scale items [10]. Dominant side was determined by asking the patients to kick a ball or which hand they prefer for writing [11, 12].

Lower extremity dexterity was assessed with the Lower-Extremity Dexterity Task (LEDT). A 3 × 3 square matrix was drawn on the floor. The dimensions of each cell were as follows: 17 cm in width and 15.3 cm in length. The central point of each cell was marked. A starting point was pointed outside of the matrix to place the participant’s feet. Participants sat on a chair adjacent to the matrix. Participants were instructed to touch each cell with their foot and then return it to the starting zone, progressing from the bottom-left to the top-right cell in an “S-shaped” sequence. After reaching the top-right cell, the same pattern was repeated in reverse order. The total time required to complete the task was recorded [4].

Fall risk was determined by retrospectively evaluating the number of falls experienced in the last six months. Participants were classified as non-fallers (no falls in the past six months) or fallers (one or more falls during the same period) [13].

Fear of falling was evaluated using the Activities-Specific Balance Confidence Scale (ABC). The ABC consisted of 16 items, and total scores range from 0 to 100. Higher scores indicate lower fear of falling. The reliability of the Turkish version of the ABC with older adults was found to be excellent (ICC = 0.997) [14].

Walking speed was assessed with the 10-Meter Walk Test (10MWT). The 10MWT requires participants to walk at a comfortable speed over a 10-m distance. The time taken to perform the test is recorded [15].

The Timed Up and Go Test (TUG) was used to assess functional walking performance. Participants were instructed to stand up from a standard chair, walk three meters, execute a 180° turn, return to the chair along the same path, and sit down [16].

Curved walking ability was assessed with the Figure-of-Eight Walk Test (FEWT). The FEWT requires participants to walk in a figure-of-eight shape between two cones placed 5 feet apart. The time taken to perform the test is recorded [17].

Backward walking ability was assessed with the Three-Meter Backward Walk Test (TMBWT). TMBWT requires participants to walk backward along a three-meter path, and the total time taken to complete the task is recorded [18].

Disease severity was assessed using the Hoehn & Yahr scale (H&Y). H&Y ranges from 1 to 5, and the higher stage indicates more severe symptoms of PD [19].

Motor symptoms were assessed using the Unified Parkinson’s Disease Rating Scale (UPDRS) Part III [20].

### Statistical analysis

Statistical analyses were performed by using IBM SPSS Statistics version 21.0 (IBM Corp. Armonk, NY). Variables were expressed as mean ± standard deviation for normally distributed data or as percentage for categorical data. The distribution of the variables was examined for normality using the Shapiro–Wilk test.

The differences between the increased fallers and non-fallers were compared using the Student’s t-test and the Mann–Whitney U test. Correlations were evaluated with Pearson and Spearman tests. Correlation coefficients were interpreted as negligible (0–0.30), low (0.31–0.50), moderate (0.51–0.70), high (0.71–0.90), or very high (0.91 or above) [21].

A logistic regression analysis was conducted to investigate the relationship between lower extremity dexterity and fall risk. In addition, the Receiver Operating Characteristic (ROC) curve analysis was used to determine the cut-off value of the LEDT. The area under the curve (AUC) is used to identify the probability of the LEDT correctly discriminating between fallers and non-fallers. The AUC was classified as follows: 1.0–0.9 = excellent, 0.89–0.80 = good, 0.79–0.70 = fair, 0.69–0.60 = poor, and 0.59–0.50 = worthless [22]. The level of significance was set as *p* < 0.05.

## Results

Thirty PwPD included in the study. The demographic and clinical characteristics of the participants are presented in Table 1. The mean age of participants was 65.53 ± 6.53 years. Eleven (36.7%) participants were female and 18 (60%) of participants were non-fallers.

**Table 1:** Demographic and clinical characteristics of the participants

	PwPD (n:30)
Age (years)	65.53 ± 6.53
Gender	
Female (%)	11 (36.7)
Male (%)	19 (63.3)
BMI (kg/m^2^)	25.81 ± 3.84
Years of education (years)	7.63 ± 3.27
Disease duration (years)	4.17 ± 2.38
H&Y (scale)	2.47 ± 0.57
Dominant side	
Right (%)	28 (93.3)
Left (%)	2 (6.7)
Affected side	
Right (%)	18 (60)
Left (%)	12 (40)
Fall status	
Fallers (%)	12 (40)
Non-fallers (%)	18 (60)
UPDRS-3 (scale)	31.47 ± 9.91
LEDT-Most affected side (seconds)	26.58 ± 8.51
LEDT-Least affected side (seconds)	24.18 ± 6.89
ABC (scale)	72.83 ± 25.14
10MWT (seconds)	7.04 ± 1.61
TUG (seconds)	10.49 ± 3.49
FEWT (seconds)	8.90 ± 2.86
TMBWT (seconds)	8.44 ± 2.30

*PwPD* People with Parkinson’s disease; *H&Y* Hoehn & Yahr scale; *UPDRS* Unified Parkinson’s disease rating scale; *LEDT* lower-extremity dexterity task; *ABC* activities-specific balance confidence scale; *10MWT* 10-m walk test; *TUG* timed up and go test; *FEWT* figure-of-eight walk test; *TMBWT* three-meter backward walk test

Comparisons of fallers and non-fallers were given in Table 2. Fallers were significantly worse than non-fallers in terms of disease duration (*p* < 0.001), LEDT-Most affected side (*p* = 0.001), LEDT-Least affected side (*p* = 0.001), ABC (*p* < 0.001), 10MWT (*p* < 0.001), TUG (*p* = 0.004), FEWT (*p* = 0.010) and TMBWT (*p* = 0.002), H&Y (*p* < 0.001), UPDRS-3 (*p* < 0.001).

**Table 2:** Comparisons of fallers and non-fallers

	Fallers (n:12)	Non-fallers (n:18)	p
Age (years)	67.58 ± 5.48	64.16 ± 6.95	0.164
Gender			
Female (%)	4 (33.3)	7 (38.9)	0.756
Male (%)	8 (66.7)	11 (61.1)	
BMI (kg/m^2^)	25.21 ± 2.90	26.20 ± 4.38	0.496
Years of education (years)	8.25 ± 3.82	7.22 ± 2.90	0.409
Disease duration (years)	6.00 ± 1.76	2.94 ± 1.92	< 0.001
H&Y (scale)	2.92 ± 0.29	2.17 ± 051	< 0.001
Dominant side			
Right (%)	12 (100)	16 (88.9)	0.144
Left (%)	0 (0)	2 (11.1)	
Affected side			
Right (%)	8 (66.7)	10 (55.6)	0.541
Left (%)	4 (33.3)	8 (44.4)	
UPDRS-3 (scale)	40.67 ± 5.14	25.33 ± 7.14	< 0.001
LEDT-Most affected side (seconds)	32.37 ± 8.57	22.71 ± 6.03	0.001
LEDT-Least affected side (seconds)	28.85 ± 6.35	21.07 ± 5.42	0.001
ABC (scale)	50.99 ± 23.22	87.4 ± 12.95	< 0.001
10MWT (seconds)	8.47 ± 0.94	6.09 ± 1.21	< 0.001
TUG (seconds)	12.64 ± 4.03	9.06 ± 2.21	0.004
FEWT (seconds)	10.49 ± 2.91	7.84 ± 2.34	0.010
TMBWT (seconds)	9.96 ± 2.21	7.42 ± 1.78	0.002

*H&Y* Hoehn & Yahr scale; *UPDRS* Unified Parkinson’s disease rating scale; *LEDT* lower-extremity dexterity task; *ABC* activities-specific balance confidence scale; *10MWT* 10-m walk test; *TUG* timed up and go test; *FEWT* figure-of-eight walk test; *TMBWT* three-meter backward walk test

The LEDT-Most affected side was found highly correlated with ABC (r = −0.755, *p* < 0.001), TUG (r = 0.775, *p* < 0.001), and FEWT (r = 0.827, *p* < 0.001) and moderately correlated with H&Y (r = 0.579, *p* = 0.001), UPDRS-3 (r = 0.684, *p* < 0.001), 10MWT (r = 0.608, *p* < 0.001), TMBWT (r = 0.692, *p* < 0.001). However, the correlation between LEDT-Most affected side and disease duration was low (r = 0.473, *p* = 0.008). Similarly, the LEDT-Least affected side was found highly correlated with ABC (r = −0.716, *p* < 0.001), TUG (r = 0.742, *p* < 0.001), and FEWT (r = 0.791, *p* < 0.001) and moderately correlated with H&Y (r = 0.608, *p* < 0.001), UPDRS-3 (r = 0.698, *p* < 0.001), 10MWT (r = 0.631, *p* < 0.001), and TMBWT (r = 0.660, *p* < 0.001). The correlation between LEDT performance on the least affected side and disease duration was modest (r = 0.486, *p* = 0.006). Due to multicollinearity, these variables were excluded from the logistic regression.

Logistic regression outcomes are summarized in Table 3. The model achieved an overall classification accuracy of 80% for distinguishing fallers from non-fallers on both the most affected and least affected sides. LEDT performance emerged as a significant independent predictor of fall risk, with an odds ratio of 1.204 (95% CI: 1.042–1.391, *p* = 0.012) for the most affected side and 1.245 (95% CI: 1.055–1.469, *p* = 0.010) for the least affected side.

**Table 3:** Logistic regression analyses

	OR	95% CI	p
LEDT-Most affected side	1.204	1.042–1.391	0.012
LEDT-Least affected side	1.245	1.055–1.469	0.010

*LEDT* lower-extremity dexterity task; *OR* odds-ratio; *CI* confidence interval

**Fig. 1:**
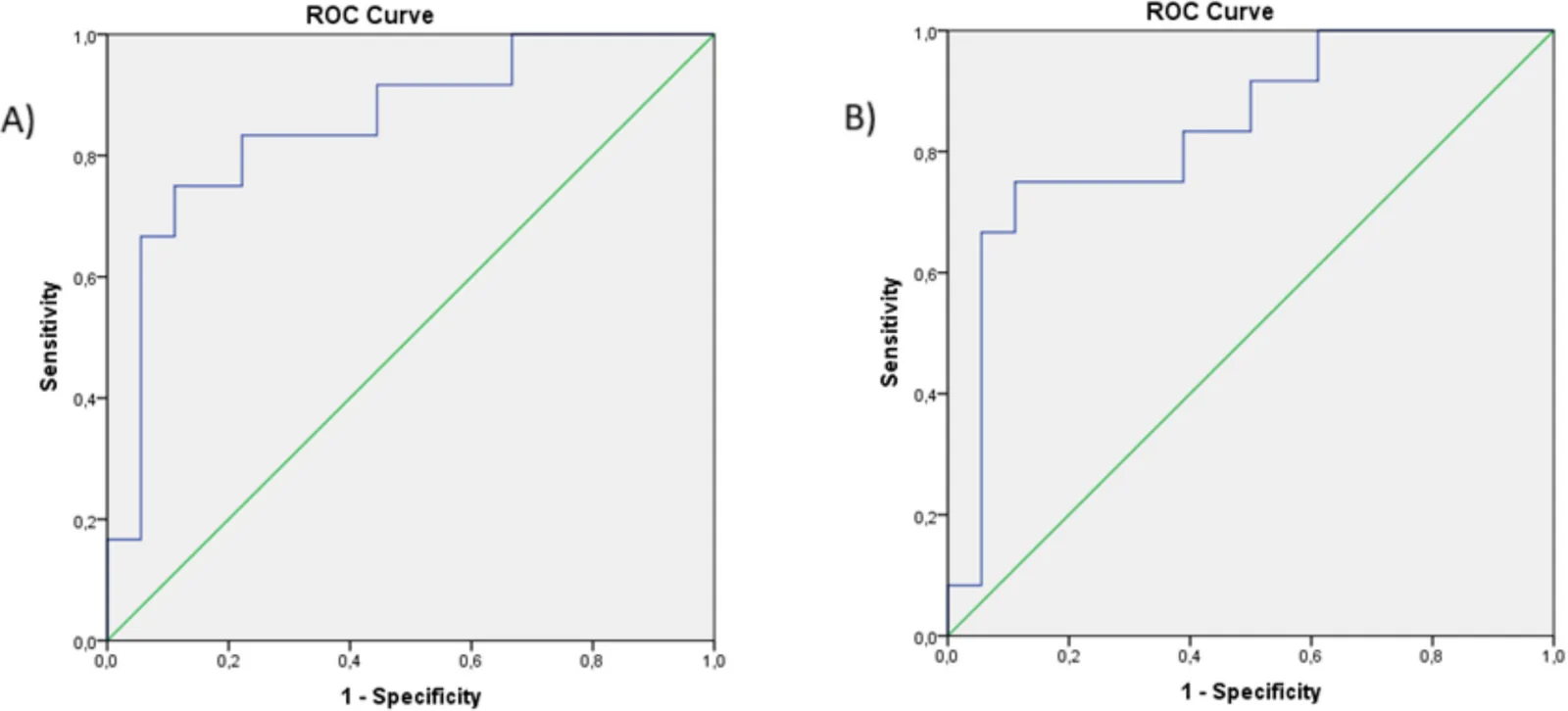
ROC analysis of the LEDT scores to discriminate fallers from non-fallers PwPD. **A**) Most affected side (cutoff time = 26.23 s; sensitivity 75.00%; specificity 88,89%; AUC = 0.852, *p* = 0.001). **B**) Least affected side (cutoff time = 23.11 s; sensitivity 75.00%; specificity 88,89%; AUC = 0.833, *p* = 0.002)

The results of the ROC analyses are shown in Fig. 1. The optimal LEDT cut-off time to discriminate fallers from non-fallers was 26.23 s (sensitivity: 75.00%; specificity: 88.89%; AUC = 0.852, *p* = 0.001) for the most affected side, and 23.11 s (sensitivity: 75.00%; specificity: 88.89%; AUC = 0.833, *p* = 0.002) for the least affected side.

## Discussion

Our results showed that 40% of PwPD have a history of falls in the last six months. Previous studies have shown that Parkinson’s disease patients experience falls ranging from 10 to 64% within a year [23–25]. However, in studies reporting falls at 6 months, this rate varies between 27 and 44% [26, 27]. Regarding fall history, our findings are consistent with previous reports.

Compared with non-fallers with Parkinson’s disease, fallers in our cohort exhibited longer disease duration, more advanced disease stage, greater motor symptom severity, poorer lower extremity dexterity, higher levels of fear of falling, reduced performance in straight, curved, and backward walking, and impaired functional mobility. Previous studies have shown that disease duration is associated with the risk of falling. Since PD is a progressive disease, it is expected that the disease will become more severe as the disease duration increases and the risk of falling will increase accordingly [28]. Similarly, worsening motor symptoms predictably increase the risk of falling. [29]. As expected, walking skills are reported to be worse in PwPD who have experienced falls [17, 18].

Functional mobility is defined as the capacity to move independently and safely within different environments to perform activities such as standing, walking, and turning [30]. These activities demand greater lower limb coordination and joint control to enable continuous postural adjustments to a changing base of support compared with straight-line walking [31]. The ability to effectively control and coordinate lower extremity movements is, by definition, referred to as lower extremity dexterity [4]. This study aimed to examine the relationships between lower extremity dexterity and fall risk, fear of falling, and walking skills in PwPD. To date, no studies have directly examined the relationship between lower extremity dexterity and fall risk in PwPD. However, previous evidence indicates a clear association between lower extremity dexterity and walking skill [32]. Accordingly, PwPD who exhibit impaired walking ability are more likely to demonstrate reduced lower extremity dexterity. Overall, 40% of PwPD in our sample reported a fall in the previous six months. Lower extremity dexterity emerged as an 80% accurate predictor of fall risk. Dexterity was also strongly associated with fear of falling, functional mobility, and curved walking skill. However, the association between lower extremity dexterity and both straight and backward walking skill was moderate. Taken together, these findings suggest that lower extremity dexterity demonstrates a stronger association with complex gait tasks demanding advanced motor control, while its relationship diminishes with less complex gait activities. Moreover, curved walking is an asymmetric action that requires more complex control of the lower extremities than straight walking [17]. Therefore, functional mobility and advanced walking skills such as curved walking, which require more lower extremity coordination and control, can be expected to be more highly related to lower extremity dexterity. Although backward walking requires more sensory-motor integration skills than forward walking, one might expect it to be less related to lower extremity dexterity since both types of walking require walking in a straight line. [18]. On the other hand, functional mobility requires complex tasks such as turning [16]. Therefore, strong relationship observed between lower extremity dexterity and functional mobility can be explained.

We also found that lower extremity dexterity was moderately associated with disease stage and severity of motor symptoms. However, its association with disease duration was weak. When the motor symptoms evaluated with UPDRS are examined, it is observed that not all of these symptoms are expected to affect lower extremity dexterity, and that the symptoms that may affect lower extremity dexterity may be observed with less severity in the lower extremities [20]. A similar relationship is predicted to be observed in the context of disease stage. [19]. Previous studies have shown that disease progression may differ substantially among PwPD who have comparable disease durations [33]. Moreover, age at disease onset has been reported to be a more influential determinant of disease progression than disease duration in some studies [34]. These factors may account for the weak association observed between disease duration and lower extremity dexterity in our findings.

Gait speed is a well-established independent predictor of fall risk in individuals with Parkinson’s disease [35]. Previous studies have also demonstrated a significant association between lower extremity dexterity and gait speed in this population [4]. In other neurological diseases, such as multiple sclerosis, it has been observed that lower extremity dexterity and balance skills are related. As mentioned before, fear of falling is an independent predictor of falls in PwPD [29]. It is reported that lower extremity dexterity and fear of falling are also related in multiple sclerosis [8]. Moreover, lower extremity dexterity is required for controlling dynamic interactions between the lower limbs and ground [36]. Robust indirect evidence indicates that lower limb motor control, gait, and balance are closely linked to cognitive function. Moreover, cognitive–motor network dysfunction may be present from the early stages of PD [37]. In this context, the finding that lower extremity dexterity predicts falls complements established predictors such as gait speed and cognitive impairment. This finding indicates that dexterity may be indicative of the neuromuscular and cognitive–motor integration necessary for ensuring safe ambulation. The findings suggest that lower extremity dexterity exhibits 80% predictive accuracy, thus may serve as a practical clinical marker of fall risk in Parkinson’s disease. The incorporation of brief dexterity assessments alongside gait speed and cognitive screening appears to enhance the evaluation of fall risk.

Our findings demonstrated a strong association between reduced lower extremity dexterity and greater fear of falling. Fear of falling is an independent predictor of falls in PwPD, and it is expected that patients with a history of falling will have a higher fear of falling [38]. It is plausible that individuals who perceive their lower limbs as poorly coordinated develop heightened fear of falling, while fear itself may further impair motor performance through overly cautious movement strategies. This bidirectional relationship indicates that improvements in lower extremity dexterity may lead to both enhanced gait performance and a reduction in fear of falling.

## Limitations

This study has several limitations. First, the fall history of participants was determined retrospectively. Therefore, the results may be misleading in terms of predicting fall risk for future falls. Second, all assessments of PwPD were performed in an “on” medication state. Therefore, it may be inaccurate to conclude the “off” medication state from our findings. Third, the study population predominantly comprised individuals with mild to moderate Parkinson’s disease; therefore, the generalizability of these findings to patients with more advanced stages of the disease may be limited. Fourth, a relatively small number of people who fall in this study may affect our results. Therefore, a larger sample size is needed.

## Conclusion

In conclusion, lower extremity dexterity emerges as an independent predictor of fall risk in PwPD. Lower extremity dexterity is also significantly associated with fear of falling and walking skill. Notably, its relationship is stronger with complex gait tasks that require advanced motor control. Therefore, it may be beneficial to implement exercises designed to enhance lower extremity dexterity to improve advanced walking skills in individuals diagnosed with PD. Further research into the relationship between lower extremity dexterity and prospective falls could yield useful results. Additionally, there appears to be a need for studies comparing lower extremity dexterity in different motor subtypes of Parkinson’s disease and between on–off periods. Furthermore, the relationship between lower extremity dexterity and dual tasking is another subject that needs investigation.

## Data Availability

The datasets generated and/or analyzed during the current study are not publicly available due to patient privacy and ethical restrictions, but are available from the corresponding author on reasonable request and with permission of the relevant ethics committee.
